# B-Cell Depletion Therapy in Systemic Sclerosis: Experimental Rationale and Update on Clinical Evidence

**DOI:** 10.1155/2011/214013

**Published:** 2011-08-03

**Authors:** Dimitrios Daoussis, Stamatis-Nick C. Liossis, Georgios Yiannopoulos, Andrew P. Andonopoulos

**Affiliations:** Division of Rheumatology, Department of Internal Medicine, Patras University Hospital, University of Patras Medical School, Rion, 26504 Patras, Greece

## Abstract

Systemic sclerosis (SSc) is a systemic rheumatic disease with poor prognosis since therapeutic options are limited. Recent evidence from animal models suggests that B-cells may be actively involved in the fibrotic process. B-cells from tight skin mice, an animal model of scleroderma, display a “hyperresponsive” phenotype; treatment with rituximab (RTX) significantly attenuates skin fibrosis in this animal model. In humans, B-cell infiltration is a prominent finding in most lung biopsies obtained from patients with SSc-associated interstitial lung disease. Several open label studies have assessed the clinical efficacy of RTX in SSc. In most patients skin fibrosis improved; lung function either improved or remained stable. Definite conclusions regarding the clinical efficacy of RTX in SSc cannot be drawn but further exploration with a multicenter, randomized study is warranted.

## 1. Introduction

Systemic sclerosis (SSc) is a systemic rheumatic disease characterized by vasculopathy, autoimmunity, and fibrosis. The available therapeutic options are extremely limited and prognosis is variable. Cyclophosphamide (CYC) has shown modest efficacy in the treatment of SSc-associated interstitial lung disease (ILD) [1] but its long-term use is accompanied by significant toxicity. Therefore, novel therapeutic approaches are desperately needed. During the last decade, B-cell depletion by rituximab (RTX), a monoclonal antibody that targets B-cells, has emerged as a promising therapy for a wide range of systemic autoimmune diseases. It has been approved for the treatment of rheumatoid arthritis but it has also been tried in systemic lupus erythematosus [2], systemic vasculitides [3], and multiple sclerosis [4], among others. An expanding body of experimental evidence suggests that B-cells play a role in the fibrotic process, raising the question of whether B-cell depletion might be a potential therapeutic approach in SSc [5–8]. During the last 2 years, 4 small-scale, open-label studies and a few case reports have addressed this question to some extent, reporting encouraging results. In this paper we provide the experimental evidence supporting the active role of B-cells in fibrosis and summarize all the available clinical evidence regarding the use of RTX in patients with SSc.

## 2. Methods

We performed a literature search in PubMed from 1995 and onwards. We used the following key words: systemic sclerosis, scleroderma, rituximab, B-cells, fibrosis, ILD, and therapy in various combinations.

## 3. Results

### 3.1. The Role of B-Cells in Fibrosis: Experimental Evidence

#### 3.1.1. Animal Models

Research in SSc has been problematic due to the low prevalence of the disease and the lack of an animal model that possesses all features of the human disease. Nevertheless, the tight skin mouse (TSK) and the bleomycin (BLM) induced mouse model of SSc have been extensively used as animal models of the disease. TSK mice are characterized by extensive skin fibrosis and immunological abnormalities including the presence of autoantibodies to topoisomerase-1, both reminiscent of those observed in the human disease [9]. A lot of research has been done on the potential role of B-cells in this animal model. It has been reported that TSK B-cells exhibit enhanced CD19 signaling compared to WT B-cells, although the expression of this molecule was similar in TSK and WT B-cells [10]. CD19 is a membrane glycoprotein of the immunoglobulin superfamily and part of the hetero-oligomeric complex comprising the complement receptor type 2, which positively regulates BCR activation. The authors reported enhanced CD19 tyrosine phosphorylation by 45% compared to WT B-cells. Tyrosine phosphorylation of Vav and Lyn kinase, both of which are important downstream steps of CD19 signaling, was also found to be enhanced by 3.4-fold and 22% respectively, compared to WT B-cells. Cytoplasmic Ca^+2^ responses, generated by CD19 ligation, were also significantly augmented in TSK B-cells compared to WT B-cells. The crucial role of CD19 signaling in this animal model is underscored by the fact that CD19 deficiency normalizes the “hyper responsive” phenotype of TSK B-cells and leads to a significant improvement of skin fibrosis compared to control TSK mice. The above data suggest that B-cell hyperactivity and fibrosis are somehow linked in this animal model and raise the question of whether targeting B-cells might be an effective way of attenuating fibrosis.

 In another study by Asano et al., the effect of enforced CD19 overexpression on B-cells in the TSK mouse model was assessed [11]. CD19 transgenic TSK mice exhibited increased autoAb production compared to control TSK mice. More specifically, CD19 transgenic mice that expressed 20% higher levels of CD19 (CD19 TG4^+/−^) and 200% higher levels (CD19 TG1^+/+^) exhibited increased antitopoisomerase-1 levels by 7.9-fold and 20-fold, compared to control TSK mice, respectively. Despite this increase in autoAb production, CD19 overexpression did not lead to worsening of skin fibrosis compared to control TSK mice. The authors also focused on gaining further insight into the hyperactive phenotype of the TSK B-cell and found that this can relate to a defective CD22 signaling, an important negative BCR response regulator. Upon stimulation, CD22 tyrosine phosphorylation was 58% lower in TSK B-cells compared to WT B-cells. The hypophosphorylation of CD22 coincided with the hyperphosphorylation of CD19, which led the investigators to suggest that defective CD22 signaling leads to increased CD19 signaling which is, at least partially, responsible for the hyperresponsive phenotype of the TSK B-cell. 

Recently it was reported that TSK mice have autoantibodies agaist CD22 which were found to be functional and able to attenuate CD22 activation [12]. Since CD22 inhibits B-cell activation, these autoAbs may contribute to the “overactivated” phenotype of TSK B-cells. In other words, TSK B-cells produce an autoAb that enhances their own activation, something that may have pathogenic implications.

Once the link between B-cell hyperactivity and fibrosis had been established in the TSK mouse, the next logical step would be to assess the effect of B-cell depletion in this animal model. RTX administration to newborn TSK mice led to a significant attenuation of skin fibrosis by 43% compared to control TSK mice accompanied by a significant reduction in autoantibody production [13]. When the same treatment was applied in 56-day old TSK mice, which are characterized by established skin fibrosis, skin thickening was not reduced, compared to TSK control mice. These data suggest that B-cells may be more important in the early phase of the disease but less so in established disease. It is noteworthy that B-cells are not present in the skin of TSK mice, indicating that their contribution to skin fibrosis may be indirect. Despite the improvement of skin thickening, B-cell depletion had no effect on the lung disease of TSK mice, indicating that different manifestations are probably mediated by different pathogenic mechanisms in this animal model. Another study explored the effects of B-cell survival factor (BAFF) inhibition in TSK mice and reported improvement of skin fibrosis and attenuation of autoAb production [14]. All these data underline the key role of B-cells in the fibrotic process in this animal model. 

The role of B-cells in fibrosis has also been explored in the BLM-induced mouse model of SSc. Besides skin involvement, lung fibrosis is a prominent feature of this animal model, making it suitable for the assessment of a given treatment on ILD. The investigators reported that BLM administration to CD19 knockout mice led to diminished skin thickening compared to BLM-treated WT littermates [15]. More importantly, CD19 deficiency led to attenuation of BLM-induced lung fibrosis, indicating that B-cells may be actively involved in the fibrotic process not only in the skin but in the lung as well. 

Recently, the role of CD19 during the development of pulmonary fibrosis in the BLM-induced model has been extensively assessed [16]. Mice either lacking or overexpressing CD19 were treated with intratracheal injections of BLM. It was reported that CD19 knockout mice exhibited less lung fibrosis in sharp contrast to mice overexpressing CD19 which showed augmented lung fibrosis compared to WT littermates. Interestingly, CD19 expression correlated with the number of B-cells in the bronchoalveolar lavage fluid; CD19 deficiency inhibited the accumulation of B-cells in the alveolar compartment following BLM challenge. The above data indicate that CD19 plays a crucial role in pulmonary fibrosis in this mouse model.

Research on animal models of scleroderma has provided evidence indicating a potential link between B-cell hyperactivity and fibrosis. However, we should note that SSc is a far more complex disease and therefore these data cannot be directly extrapolated to humans.

#### 3.1.2. Humans

Only a few studies have addressed the potential contribution of B-cells in the pathogenesis of SSc. In one such study, it was reported that B-cells from patients with SSc had 20% higher CD19 expression compared to B-cells from healthy subjects [17]. Detailed phenotypic characterization of B-cells from patients with SSc revealed that peripheral blood B-cells were increased in patients with SSc compared to healthy subjects [18]. Naive B-cells were reported to be increased in patients with SSc whereas memory B-cells and plasmablasts were reduced, compared to healthy subjects. Memory B-cells from patients with SSc had increased expression of several activation markers, including CD95 and were prone to spontaneous apoptosis. These data indicate that B-cell homeostasis in SSc is disturbed. 

Since patients with SSc express higher levels of CD19 on B-cells and since CD19 is a key molecule in the regulation of signaling thresholds in these cells, something that may relate to break of tolerance and induction of autoimmunity, investigators explored the mechanisms involved in CD19 overexpression in SSc B-cells. Tsuchiya et al. explored the potential association of CD19 polymorphisms with SSc and the level of CD19 expression on B-cells [19]. They reported a significant association between the −499T allele in the promoter region of the CD19 gene with SSc, with an odds ratio of 2.18; carriers of this allele exhibited significantly higher CD19 levels on B-cells compared to noncarriers. These data raise the question of whether CD19 upregulation in SSc B-cells is genetically determined.

It is not clear why SSc B-cells exhibit such an “overactivated” phenotype; B-cell survival factors may be implicated. It has been reported that BAFF serum levels are higher in patients with SSc compared to healthy controls (*P* < 0.001) [20]. Furthermore, patients with the diffuse form had significantly increased levels compared to patients with the limited form of the disease. Nevertheless, this was not a disease-specific finding, since patients with SLE or DM had similarly increased levels. Patients with SSc, exhibiting increased serum BAFF levels tended to have more severe skin fibrosis as assessed by the MRSS tool (*P* < 0.01), worse FVC values (*P* < 0.05) and higher ESR (*P* < 0.05). Furthermore, decreasing serum BAFF levels were associated with attenuation of skin fibrosis, whereas increasing levels correlated with clinical worsening. The expression of BAFF in the skin at the mRNA level was found to be increased in early disease. Moreover, upregulation of the BAFF receptor was reported on B-cells from patients with SSc compared to healthy subjects. It is also worth mentioning that B-cells from patients with SSc stimulated with BAFF produced 38% more IL-6, a cytokine able to stimulate fibroblasts, compared to B-cell from healthy subjects.

In a recent study it was found that peripheral blood mononuclear cells (PBMCs) from SSc patients produced significantly more APRIL (a proliferation inducing ligand), a B-cell survival factor, compared to PBMCs from healthy subjects (*P* < 0.01) [21]. This increase was associated with more severe disease manifestations. These data indicate that upregulation of B-cell survival factors may contribute to B-cell hyperactivity and autoimmunity in SSc. BAFF and APRIL emerge as two interesting therapeutic targets; inhibition of these molecules may modulate B-cell function in SSc and potentially lead to clinical benefit.

An interesting study, performed by Whitfield et al. examined gene expression profile, using microarrays in scleroderma skin compared to normal [22]. The authors found that genes characteristic of B-cells, fibroblasts and endothelial cells were differentially expressed in scleroderma compared to normal skin. Interestingly, the same expression pattern was evident in patients with SSc in both clinically involved as well as uninvolved skin, underscoring the systemic nature of the disease. Since endothelial cells and fibroblasts are considered key players in SSc, these data point towards a potentially active role of B-cells in skin fibrosis. 

The potential role of B-cells in SSc-associated ILD has been inadequately investigated. It has been reported that B-cells are present in lung biopsies from patients with SSc-associated ILD [23] and that plasma cell infiltration of the alveolar walls is an early finding [24].

The above studies have documented the presence of B-cells in both skin and lung of patients with SSc. However direct evidence of a pathogenetic role is lacking.

### 3.2. B-Cell Depletion in SSc: Update on Clinical Evidence

There are 4 small-scale, open-label clinical studies exploring the potential clinical efficacy of RTX in SSc, including one from our research group and a few case reports. In the first study by Smith et al., eight patients with early (defined by disease duration of <4 years from the first non-Raynaud's disease manifestation) diffuse SSc received a single course of RTX (consisting of 2 infusions, 1000 mg each, at day 1 and 15) [25]. Patients were evaluated clinically at 24 weeks and subjected to skin biopsies at baseline and 12 weeks. Five patients had evidence of mild ILD; patients with severe ILD were excluded from the study. Improvement of skin thickening was reported as assessed by the MRSS tool (mean ± SD: 24.8 ± 3.4 versus 14.3 ± 3.5 at baseline versus 24 weeks resp., *P* < 0.001). It is worth mentioning that improvement of skin thickening was verified at the histological level, since both collagen and myofibroblast score were reduced significantly following treatment (*P* = 0.014 and *P* = 0.013, resp.). Half of the patients had evidence of B-cell infiltration in their skin; treatment effectively depleted these cells. Lung function tests, systolic pulmonary artery pressure, left ventricular ejection fraction, creatinine clearance, and HAQ score remained stable throughout the study. Two serious events were reported (one patient underwent coronary artery bypass surgery and another was hospitalized due to low-grade fever that spontaneously subsided) but were considered to be probably unrelated to study drug. This is the first study that provides clinical as well as histological evidence that RTX treatment may favourably affect skin fibrosis in SSc. It should be noted however, that this study has potential limitations which are the relatively small number of patients recruited and the lack of a control arm. Resolution of skin fibrosis is associated with the natural course of the disease, therefore uncontrolled studies are difficult to interpret. Nevertheless, the improvement of skin fibrosis reported in this study was quite significant, which makes it rather unlikely to have occurred spontaneously, especially within the limited time frame of the study.

Another study reported the effects of a single course of RTX treatment in 15 patients with early (as defined by disease duration of <18 months from the first non-Raynaud's disease manifestation) diffuse SSc [26]. Similar to the study by Smith et al., only 7 out of 15 patients had evidence of mild ILD, since the existence of moderate or severe ILD was an exclusion criterion. Patients were clinically evaluated at 24 weeks and 1 year. In contrast to the study by Smith et al., no improvement of skin thickening as assessed by the MRSS, tool was reported (mean ± SD: 20.6 ± 4.4, 20.2 ± 5.5 and 21.1 ± 5.2 at baseline, 24 wks, and 48 wks, respectively, *P* = ns). However, histologic improvement was found; myofibroblast score declined from 49.5 to 36.6 (*P* < 0.05). Skin infiltrating B-cells were significantly increased in patients with SSc compared to healthy controls who had no B-cells and were eliminated posttreatment. Pulmonary function tests remained stable at 24 wks compared to baseline; it is noteworthy though that FVC values increased by an average of 3.5% at 24 wks compared to pretreatment values, but the 95% CI was wide and thus results were not statistically significant. The authors do not report PFTs at 1 year posttreatment. No evidence of new or progressive major organ involvement was reported. Treatment was well tolerated. 

Our research group has performed an open label, randomized controlled, 1-year pilot study, assessing the effect of RTX in SSc [27]. We recruited 14 patients with SSc randomized as follows: 8 patients in the treatment arm and 6 patients in the control arm of the study. All patients had diffuse disease, were anti-Scl70 positive, and had evidence of ILD. There were no differences in terms of disease duration, baseline MRSS and baseline PFT's between the treatment and the control group. Patients in the treatment arm received 2 cycles of RTX at baseline and 24 weeks (each cycle consisting of 4 weekly RTX infusions (375 mg/m^2^)). We found a significant improvement of both FVC (mean ± SD: 68.13 ± 19.69 versus 75.63 ± 19.73% of predicted values, at baseline versus 1 year resp., *P* = 0.0018) and DLco (mean ± SD: 52.25 ± 20.71 versus 62 ± 23.21% of predicted values, at baseline versus 1 year, resp., *P* = 0.017) in the treatment group whereas no change was noticed in the control group. The median (upper and lower quartile values) percentage of improvement of FVC in the RTX group was 10.25% (6.19–18.65) whereas in the control group FVC deteriorated (median percentage of deterioration (upper and lower quartile values) 5.04% (4.11–11.6)). Direct comparison of FVC changes recorded at 1 year, revealed that the RTX-treated group improved significantly (*P* = 0.002) compared to the standard-treatment (control) group. The median (upper and lower quartile values) percentage of improvement of DLco in the RTX group was 19.46% (3.7–30.8) whereas in the control group the median percentage of deterioration was 7.5% (1.4–26.57) (*P* = 0.023). 

Skin thickening, assessed with the MRSS, was similar in the two treatment groups at baseline (*P* = 0.50). However at the 1 year evaluation, there was a significant decrease of MRSS in the RTX group compared to the baseline score (mean ± SD, 13.5 ± 6.84 versus 8.37 ± 6.45 at baseline versus 1 year, resp., *P* = 0.0003). On the contrary, no significant change in skin scores was noticed in the control group at 1 year when compared to the baseline MRSS (mean ± SD, 11.50 ± 2.16 versus 9.66 ± 3.38 at baseline versus 1-year, resp., *P* = 0.16). The median (upper and lower quartile values) percentage of improvement in the RTX-treated group was 39.25% (27.33–64.95) compared to 20.80% (10.78–39.28) in the control group. Statistical analysis revealed that differences tended to be but were not statistically significant (*P* = 0.06). Improvement of skin fibrosis was also documented at the histological level. We found a significant reduction of collagen deposition in the papillary dermis at 24 wk compared to baseline in patients treated with RTX; histologic improvement correlated with skin B-cell depletion. Histological data matched the clinical data, since all patients showing histologic improvement also improved clinically. One serious adverse event was reported (respiratory tract infection); the patient recovered fully following short-term hospitalization.

Even though this is the first and only so far, randomized, controlled study assessing the efficacy of RTX in SSc, several limitations exist. Firstly, the small number of patients recruited does not provide the study with sufficient statistical power to prove efficacy. Additionally, most patients had long-standing disease since no disease duration restriction was applied and was heterogeneous in terms of disease duration, severity, and previous treatments.

Recently, one more study assessing the clinical efficacy of RTX in SSc was published [28]. Nine patients with SSc were recruited and received one course of RTX (consisting of 2 infusions, 1 gr each). All patients had severe cutaneous involvement and had experienced worsening of skin score despite treatment with CYC. A significant reduction of skin thickening was reported with patients showing a median decrease of skin score of 43.3% at 6 months compared to baseline. Disease activity and severity index also declined. PFTs remained stable throughout the study. A significant decline in IL-6 levels following treatment was also reported; the authors hypothesized that this may have contributed to attenuation of skin fibrosis. 

Three case reports have also appeared in the literature regarding the use of RTX in SSc. The first case describing the beneficial effect of RTX on SSc-associated ILD was reported by McGonagle et al. [29]. PFTs, musculoskeletal manifestations and functional status improved following treatment. However treatment effect waned over time and a second course of RTX was administered. PFTs improved again; DLco increased from 34.3% to 48% of predicted values. These data are in agreement with ours, showing that consecutive treatment courses may be needed to augment and sustain the effect of RTX on pulmonary function.

The beneficial effect of RTX on SSc-associated ILD has also been documented in a case report by our group [30]. Our patient was treated with 4 consecutive RTX courses every 6 months and completed a followup of 2 years. PFTs significantly increased; FVC and DLco reached values of 35%, and 33% respectively, compared to 30% and 14% of pretreatment values. Quantification of ground glass lesions using a computer-aided diagnosis system showed a 14% reduction. Skin thickening improved as indicated by a decline in MRSS from 20 to 9. Clinical improvement coincided with histologic improvement with reduction of collagen accumulation and myofibroblast score; skin infiltrating B-cells were eliminated post treatment. The functional status of the patient improved as indicated by a decrease in HAQ score and an increase in 6-minute walking distance. This was the first report of long-term RTX treatment in SSc. Recently, another case of successful treatment of CYC-resistant SSc-associated ILD was reported [31]. Finally, RTX seems to favourably affect ILD in the context of other systemic rheumatic diseases such as the antisynthetase syndrome as indicated by several reports [32, 33]. All the available published data regarding the clinical efficacy of RTX in SSc are summarized in Table 1.

## 4. Discussion

SSc is perhaps the most challenging disease for rheumatologists. So far treatment is based on nonspecific immunosuppression, with agents such as CYC [1] or mycophenolate mofetil [34], with modest results. RTX has been used with varying degrees of success in most systemic autoimmune diseases and this is why, one may argue that its effect on a severe, incurable systemic autoimmune disease such as SSc, is worthwhile exploring. Furthermore, there is a strong rationale for the use of RTX in SSc; an expanding body of evidence from basic research points to the direction that B-cells may be active players in the fibrotic process. On clinical grounds, until now, 43 patients with diffuse SSc have been treated with RTX (available published data). What conclusions can be made regarding the use of RTX in SSc? First of all, this agent seems to be well tolerated in SSc since only few adverse events have been reported. But is RTX clinically effective in SSc? Based on the available clinical evidence, definite conclusions cannot be drawn; however results are encouraging. In 3 out of 4 studies skin thickening significantly improved and, even in the single study where no clinical benefit on skin thickening was found, histologic improvement was documented. These data may suggest a disease modifying role of RTX in skin fibrosis. 

The most fearful manifestation of SSc is lung disease which is nowadays the leading cause of mortality. The clinical evidence on the efficacy of RTX in SSc-associated ILD is limited, since most patients recruited either did not have ILD or had only very mild ILD. With the exception of our study, the other 3 studies were not designed to test the potential clinical efficacy of RTX in SSc-associated ILD. Nevertheless, in these three studies PFTs remained stable, even though most patients had early disease and therefore were most likely to exhibit declining PFTs during their followup. In our study, a significant improvement of lung function was reported in contrast to other studies where PFTs remained stable at 6 months compared to baseline. We should note, however, several differences in the design of the studies that could potentially explain these diverse findings. In the other studies most patients did not have ILD in contrast to our study where the presence of ILD was an inclusion criterion. Furthermore, the higher dose of RTX used (4 × 375 mg/m^2^ instead of 2 × 1000 mg), consecutive treatments, and the longer evaluation period could also be potential explanations. The significant improvement in lung function tests observed in our study indicates that RTX may favourably affect SSc-associated ILD. Long-term treatment with RTX may either improve or stabilize lung function over time in patients with SSc. All 8 patients recruited in the treatment arm group of our study remained on RTX treatment and received two additional courses; their PFTs continued to improve during the second year of followup [35]. 

If RTX turns out to be an effective treatment for SSc, which could be the potential mechanisms of action? Several possibilities exist. First, B-cells appear to be actively involved in the fibrotic process, as indicated by data derived from both animal models and humans. Elimination of skin infiltrating B-cells by RTX has been documented, albeit not consistently. Taking into account that B-cell infiltration is a prominent finding in lung biopsies from patients with SSc-associated ILD, depletion of this population may be a potential explanation for the clinical improvement. It would therefore be of great interest to study the effect of RTX on lung disease at a histological level, but this may be inherently difficult and challenging. We have also recently shown that RTX-induced improvement of skin fibrosis associates with a decrease in PDGFR phosphorylation (which corresponds to activation) in scleroderma skin [36]. Finally, another mechanism may involve indirect effect(s) of RTX on other cells, such as T cells [37].

Both experimental and clinical evidence regarding B-cell depletion in SSc is encouraging and certainly points to the direction that further exploration of its clinical efficacy is warranted. The best way forward would be a multicenter, randomized, double blind, placebo-controlled study. If such a study is performed we propose that it should focus on ILD rather than skin disease and that it should be designed in such a way, that patients are treated for at least one year and evaluated thereafter; in this way, a treatment effect on ILD (if any) would be more easily depicted. To our knowledge, up until now, no such study has been registered. Interestingly, however, a phase II study assessing the clinical efficacy of RTX in SSc-associated pulmonary arterial hypertension has already been launched (http://clinicaltrials.gov/show/NCT01086540). Moreover, a phase I study of MEDI-551 (mAb against CD19) has also been launced (http://clinicaltrials.gov/show/NCT00946699). We believe that a large-scale multicenter, randomized study assessing the potential clinical efficacy of RTX in SSc is highly needed.

## Figures and Tables

**Table 1:** Studies assessing the efficacy of RTX in SSc.

							Outcomes		
						Skin		Lung function tests	Functional status
Study	Number of participants	Study type	Evaluation time point(months)	Number of RTX courses					

					Clinical assessment	histologic improvement (yes/no)	Skin B-cell depletion		
Smith et al.	8	Open label, uncontrolled	6	1	Improved	Yes	In 4 patients	Stable	Stable

Lafyatis et al.	15	Open label, uncontrolled	6/12	1	Stable	Yes	Yes	Stable	Stable

Daoussis et al.	8	Open label, randomized controlled	12	2	Improved	Yes	Yes	Improved	Improved

Boselo et al.	9	Open label, uncontrolled	6–36	1 (3 patients receive a second cycle)	Improved	Not reported	No	Stable	Improved

McGonagle et al.	1	Case report	—	2	Not reported	Not reported	—	Improved	Improved

Daoussis et al.	1	Case report	24	4	Improved	Improved	Yes	Improved	Improved

Yu	1	Case report	—	1	Not reported	Not reported	—	Improved	Improved
